# Measurement of the Induced Magnetic Polarisation of Rotated-Domain Graphene Grown on Co Film with Polarised Neutron Reflectivity

**DOI:** 10.3390/nano13192620

**Published:** 2023-09-22

**Authors:** Razan Omar M. Aboljadayel, Christy John Kinane, Carlos Antonio Fernandes Vaz, David Michael Love, Marie-Blandine Martin, Andrea Cabrero-Vilatela, Philipp Braeuninger-Weimer, Adrian Ionescu, Andrew John Caruana, Timothy Randall Charlton, Justin Llandro, Pedro Manuel da Silva Monteiro, Crispin Henry William Barnes, Stephan Hofmann, Sean Langridge

**Affiliations:** 1 Cavendish Laboratory, Physics Department, University of Cambridge, Cambridge CB3 0HE, UK; 2 ISIS Facility, STFC Rutherford Appleton Laboratory, Harwell Science and Innovation Campus, Oxon OX11 0QX, UK; 3 Swiss Light Source, Paul Scherrer Institut (PSI), 5232 Villigen, Switzerland; carlos.vaz@psi.ch; 4 Department of Engineering, University of Cambridge, Cambridge CB3 0FA, UK

**Keywords:** graphene, PNR, XMCD, magnetism, heterostructures

## Abstract

In this paper, we determine the magnetic moment induced in graphene when grown on a cobalt film using polarised neutron reflectivity (PNR). A magnetic signal in the graphene was detected by X-ray magnetic circular dichroism (XMCD) spectra at the C *K*-edge. From the XMCD sum rules an estimated magnetic moment of 0.3 μ_B_/C atom, while a more accurate estimation of 0.49 μ_B_/C atom was obtained by carrying out a PNR measurement at 300 K. The results indicate that the higher magnetic moment in Co is counterbalanced by the larger lattice mismatch between the Co-C (1.6%) and the slightly longer bond length, inducing a magnetic moment in graphene that is similar to that reported in Ni/graphene heterostructures.

## 1. Introduction

Spintronics is one of the fast-growing fields for realising next-generation technologies [1,2]. Spintronic devices, which utilise the two properties of the electron (charge and spin degrees of freedom), hold promises for solving the issues of conventional electronics by providing non-volatile, fast and ultra-low heat dissipation technologies [2,3]. Therefore, many studies focus on finding materials that can be used to easily generate, manipulate and detect spins [3,4].

In particular, two-dimensional (2D) materials have unique properties making them potential candidates for spintronics applications. However, there are still several challenges to be overcome to realise 2D spintronics devices. For instance, most 2D materials are not ferromagnetic (FM). Therefore, they require special spin injection approaches, which could be difficult due to their reduced thicknesses [4].

One of the possible solutions for the current limitations of 2D materials is to stack different materials in specially-engineered heterostructures, which would allow one to utilise the best properties of each material and combine them into one unique structure. This has been made possible by recent advancements in deposition and measuring techniques which have in turn opened new research areas in the field [4].

Graphene has seen a breadth of applications so far ranging from metrology, where it is currently used as an electrical resistance standard by means of the quantum Hall effect [5], to applications in bio-sensors based on surface plasmon resonance (SPR) [6]. Further possible applications of graphene based SPR have been proposed for tunable terahertz absorbers recently [7,8]. Graphene is also one of the most promising materials for 2D spintronics. It has a long spin diffusion length and large spin relaxation lifetime due to its small spin-orbit and hyperfine couplings [9,10]. Furthermore, manipulating spins directly in graphene has been achieved via various methods such as the proximity-induced effect when it is grown on top of a ferromagnetic material [9,11,12,13,14]. The strong interaction between a lattice-matched FM and the graphene distorts the graphene’s band structure which opens the its Dirac cone as a result of the breaking of degeneracy around the Dirac points inducing magnetism in the graphene. This has been explained in the universal model proposed by Voloshina and Dedkov [15] which was proved theoretically and experimentally using density functional theory calculations and angle-resolved photoemission spectroscopy, respectively [15,16,17,18,19,20,21].

In a previous study, we determined quantitatively the induced magnetic moment in epitaxial and rotated-domain graphene grown on Ni films using polarised neutron reflectivity (PNR) [22]. Although the interaction between the Ni and rotated-domain graphene was expected to be weaker compared to that with epitaxial graphene due to the missing epitaxial relationship, we found that a similar magnetisation was induced in both structures. In this study, we investigate the effect of the magnetic moment amplitude of the FM layer on the induced magnetisation in the graphene layer by using Co as the ferromagnetic layer. Co has a higher magnetic moment of ∼$1.7\;\mu_{B}$/atom as compared to $0.6\;\mu_{B}$/atom for Ni, a slightly larger lattice mismatch of ∼1.6% (∼1.2% in Ni) to graphene and a bond length of 2.16 Å compared to Ni (2.03 Å) [15,17,18,23]. For a review on the growth of graphene on various transition metal catalysts see Ref. [24]. Furthermore, epitaxial single layer graphene grown on a Co single crystal and multilayer-rotated graphene grown on polycrystalline Co layers have also been previously reported in Refs [25,26].

In this paper, we discuss the growth conditions of the Co and graphene and present the structural properties of the sample measured by X-ray diffraction, X-ray reflectivity and scanning electron microscopy. The quality of the graphene was determined by Raman spectroscopy, whereas X-ray magnetic circular dichroism (XMCD) and PNR measurements were conducted to determine quantitatively the magnetic moment in graphene and Co separately.

## 2. Materials and Methods

The Co film was grown at room temperature (RT) on a 1 mm thick Al${}_{2}$O${}_{3}$(0001) substrate using a Surrey Nanosystems magnetron sputtering chamber (with a base pressure of ∼1.5 × 10${}^{-8}$ mTorr), using 99.9% pure Co target. The growth conditions (a DC current of 0.1 A, a pure argon flow of 14 sccm and a plasma pressure of 2 mTorr) were maintained throughout the deposition to grow 80 nm of highly textured hcp Co(0001) film at a rate of 0.02 nm/s.

Rotated-domain graphene was then grown directly on the Co layer using a Aixtron Black Magic Pro chemical vapour deposition (CVD) system (Aixtron SE, Herzogenrath, Germany). During the process, H${}_{2}$ gas was first injected into the chamber (with a base pressure of 2.7 × 10${}^{-6}$ mbar) at a rate of 180 sccm. The temperature of the growth chamber was then ramped at 100 ${}^{\circ }$C per min to 630 ${}^{\circ }$C and annealed for 12 min. The sample was then subsequently exposed to C${}_{2}$H${}_{4}$ with a flow rate of 0.63 sccm for 60 min and then left to cool down in a vacuum to RT. This method was adapted to eliminate the presence of any oxidised Co surface by reducing it back to Co film before growing the graphene.

RT X-ray diffraction (XRD) measurements were performed using a Bruker D8 Discover HRXRD (Bruker, Billerica, MA, USA) with a Cu Kα monochromatic beam (voltage of 40 kV and a current of 40 mA) to study the crystallinity of the Co film, while for estimating the thickness, roughness and density of the film X-ray reflectivity (XRR) measurements were performed with a Cu rotating anode 9 kW Rigaku Smartlab diffractometer (Rigaku Corp., Tokyo, Japan) with a beam voltage of 45 kV and a current of 200 mA. A FEI Magellan 400L scanning electron microscope (SEM) (previously FEI, now Thermo-Fisher Scientific, 5350 NE Dawson Creek Drive, Hillsboro, OR, USA) was used to study the homogeneity, structure and coverage of the rotated-domain graphene. Furthermore, Raman spectroscopy measurements were carried out using a Renishaw InVia spectrometer (Renishaw plc, Wotton-under-Edge, UK) to investigate the quality of the graphene. An excitation laser with a wavelength of 532 nm, spot size of ∼1μm, 10% laser power, 100× objective lens and exposure time of 0.5 s was used to scan three different regions of the sample.

The bulk magnetic properties of the sample were investigated using a Quantum Design superconducting quantum interference device (Quantum Design, San Diego, CA, USA). X-ray magnetic circular dichroism (XMCD) was used to distinguish the magnetic signal of the graphene layer from that of Co. Room temperature (300 K) measurements using total electron yield (TEY) mode with 100% circularly polarised light were performed at the SIM beamline at the Swiss Light Source (SLS) at the Paul Scherrer Institut (PSI), Switzerland. An experimental setup similar to that reported in Ref. [22] was adopted to measure the Gr/Co sample.

Furthermore, polarised neutron reflectivity (PNR) measurements were conducted to provide a more accurate estimation of the magnetic moment induced in graphene. These measurements were taken at the POLREF beamline [27] at ISIS pulsed spallation neutron source, Rutherford Appleton Laboratory, Didcot, UK. The sample was measured at 300 K with an in-plane applied field of 0.5 T using a similar setup to that reported in Ref. [22] to measure graphene grown on Ni and Ni${}_{9}$Mo${}_{1}$ films. The *Refl1D* software v0.8.16 [28] package was used to fit the data, based on the analysis methodology described in Ref. [22].

## 3. Results and Discussion

### 3.1. X-ray Diffraction and X-ray Reflectivity

**Figure 1:**
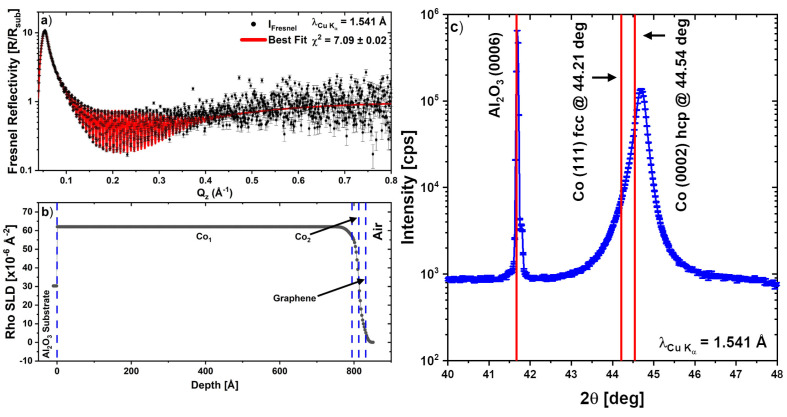
(**a**) Room temperature XRR measurements of Graphene/Co/Al${}_{2}$O${}_{3}$(0001). (**b**) SLD profile as derived from the XRR fit. (**c**) X-ray diffraction measurements of an as-grown highly textured hcp Co(0001) film on Al${}_{2}$O${}_{3}$(0001). The vertical red lines are the bulk peak positions.

Figure 1a shows the XRR results together with the corresponding fit to the data with the scattering length density (SLD) profile shown in panel (b) as derived from the XRR fit. The fitting parameters (thickness (z), roughness (σ) and density (ρ)) of each layer of the sample were used as starting parameters for the PNR fits and are summarised in Table 1. It should be noted that the roughness of the graphene layer is coupled to that of the top cobalt layer as described in the modelling for the Ni/Graphene samples in Ref. [22]. It is also noteworthy to mention that, like in the case of the Ni/Gr in Ref. [22], there are large residuals at and below the critical edge which are suspected to be due to the footprint correction not capturing the full geometry of the X-ray beam footprint as it changes on the sample. The sample was a non-square shard, which exacerbates this issue. Hence the XRR is used only as a starting point for the PNR analysis. On the other hand, the PNR is a time-of-flight technique with a fixed footprint and, as such, does not suffer from systematic errors due to the interaction of the beam footprint and the sample shape.

The XRD scan presented in Figure 1c shows a highly textured Co film oriented we suspect in the hcp [0001] direction. We only obtained one peak from the Co layer in the XRD scan, hence we cannot determine from that alone whether this is fcc Co, with a (111) reflection (2θ = 44.21 deg), or hcp Co, with a (0002) reflection (2θ = 44.54 deg). These positions are shown as solid red vertical lines in Figure 1c. Our peak is at 44.69 degrees in 2θ which is well aligned with the Al${}_{2}$O${}_{3}$ (0006) peak at 41.68 degrees. We cannot rule out that substrate strain due to epitaxy has stabilised the fcc phase in the 80 nm thick film. However, based on the fact the peak is closer to the hcp value which is also the stable low-temperature phase, fcc being the high-temperature phase above about 700 K, we suspect we have hcp Cobalt grown to the Al${}_{2}$O${}_{3}$ substrate.

### 3.2. Scanning Electron Microscopy

**Figure 2:**
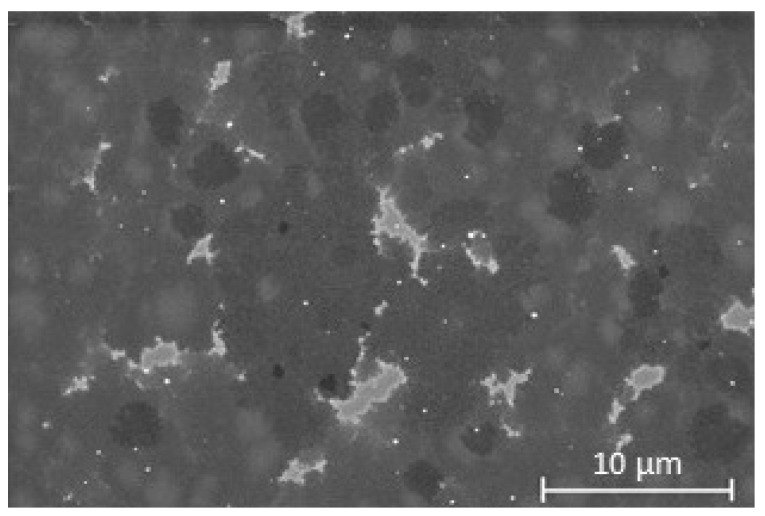
SEM image taken at 2 kV accelerating voltage showing the graphene domains for graphene grown on a Co film, where the light grey region is bare Co and different shades of darker grey suggest mixed areas of single, bi- and multi-layer graphene.

Figure 2 shows an SEM image of the differently oriented graphene domains on the Co film with a surface coverage of ∼90%. The light grey region is bare Co, whereas the different shades of darker grey suggest mixed areas of single, bi- and few-layer graphene grown on Co.

### 3.3. Raman Spectroscopy

Raman spectroscopy was used to examine the quality and to estimate the number of graphene layers, doping and defect density in the grown graphene. Three different scans at different spots on the sample surface were taken in the range between 1200 cm${}^{-1}$ to 2800 cm${}^{-1}$ to observe the main characteristic peaks of graphene; G and 2*D* peak at ∼1582 cm${}^{-1}$ and ∼2700 cm${}^{-1}$, respectively, as well as the second-order Raman scattering peaks; *D+D*″ and 2*D*′ peaks at ∼2450 cm${}^{-1}$ and ∼3200 cm${}^{-1}$, respectively, and to check for the presence of any disorder-induced peaks such as *D* and *D*′ peaks at ∼1350 cm${}^{-1}$ and ∼1600 cm${}^{-1}$, respectively [30,31,32,33].

Before carrying out these scans, the Co film was chemically etched with a 20% HNO${}_{3}$ solution in order to transfer the graphene onto a Si/SiO${}_{2}$ (300 nm) substrate in a similar manner to that reported in Ref. [34]. This is because the strong chemical interaction between the graphene and the closely-lattice match Co film alters the Raman resonance conditions of graphene. Furthermore, the extension of the graphene’s C-C bond length to match that of Co alters the graphene’s phonon spectrum [18,31].

**Figure 3:**
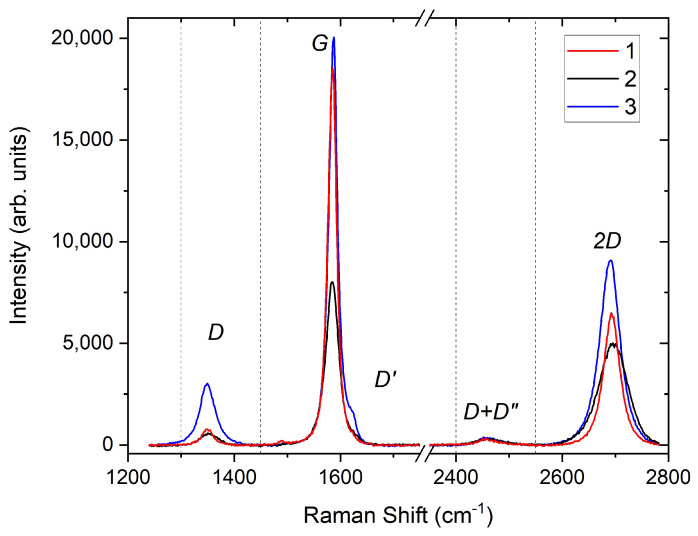
Raman spectroscopy measurements taken at 300 K at three different regions of graphene transferred to a Si/SiO${}_{2}$ substrate, showing the graphene’s characteristic peaks. The dashed vertical lines separate the regions of the different peaks.

The RT Raman scans of the transferred graphene is shown in Figure 3. The shape of the spectra confirms the spatial inhomogeneities in our sample given the laser spot size of ∼1μm and the SEM image in Figure 2 showing variations in the flake size and thickness on the range of a few micrometre. These inhomogeneities could be intrinsic to the growth of graphene on Co, which has a high carbon solubility as compared to Cu for example [25], and could have been further exacerbated by the chemical etching and transfer process to the Si/SiO${}_{2}$ substrate. Although all the 2*D* peaks shown in Figure 3 were fitted with single Lorentzians, they have a relatively broad full width at half maximum (FWHM). The large FWHM could be attributed to a high defect density, or to doping from the HNO_3_ used to etch the metallic films. The average FWHM of the 2*D* peak for spectrum 1 and 2 is 40.11 cm${}^{-1}$, whereas the FWHM for spectrum 3 is 63.19 cm${}^{-1}$ which confirms the spatial inhomogeneity also seen in the SEM scan (Figure 2). The average *I${}_{2D}$*/*I${}_{G}$* ratio of 0.47 (i.e., <1.5) and the 2*D* peaks with FWHM ∼50 cm${}^{-1}$ suggest a few-layer graphene system [24]. Nevertheless, the very low average *I${}_{D}$*/*I${}_{G}$* ratio (0.09) and the fact that only the blue spectrum (number 3) has a small *D*′ shoulder suggest that good-quality few-layer graphene with low defect density was grown on the Co film. These results are consistent with the SEM image and the thickness estimated from the XRR fits. Although a few-layer graphene system was grown on Co, we can infer that they are less than five layers in total since it was reported previously that only the Raman spectrum of graphene with up to five layers could be distinguished from that of graphite [35].

### 3.4. X-ray Magnetic Circular Dichroism

**Figure 4:**
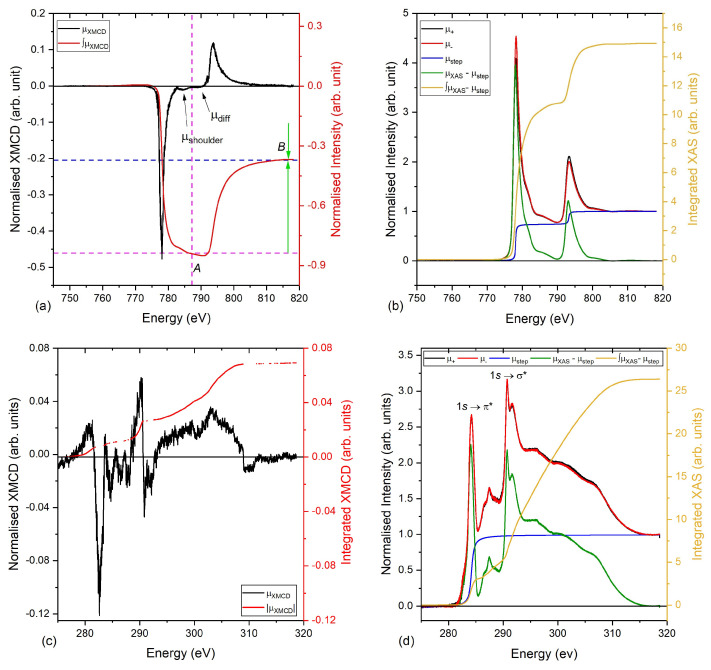
X-ray magnetic circular dichroism and X-ray absorption spectra with the regions used to apply the sum rules for the graphene/Co sample, measured at 300 K: (**a**,**b**) XMCD and XAS spectra for the Co *L*_2,3_-edge; (**c**,**d**) XMCD and XAS spectra for the graphene layer.

The XMCD and X-ray absorption spectra (XAS) of the graphene/Co system measured at 300 K are shown in Figure 4. The sample was fixed at an incident angle of 30${}^{\circ }$ from the incident X-ray beam, to enhance the magnetic contrast at the C $\pi^{*}$ resonance. A magnet, fixed at 40${}^{\circ }$ to the incident X-ray beam, was used to apply an in-plane field of 0.11 T to magnetise the Co film. After 30 s, the field was reduced to 0.085 T for the XAS and XMCD measurements.

**Figure 5:**
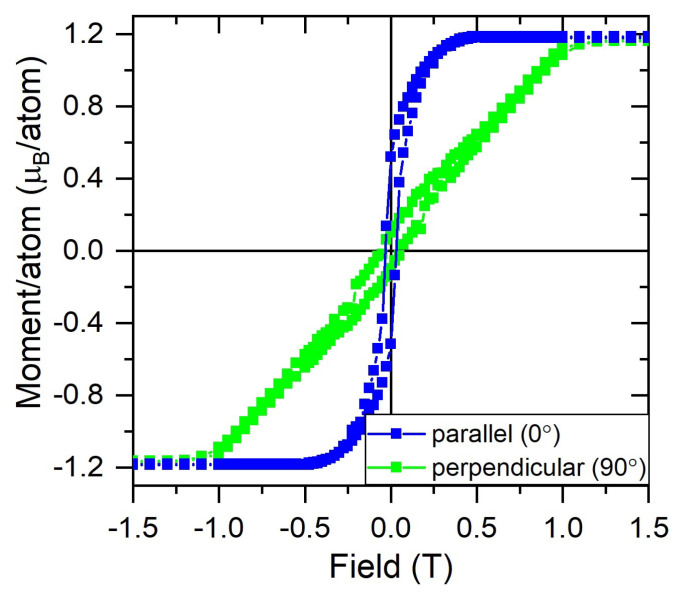
SQUID measurements at 300 K showing the hysteresis loops obtained with the magnetic field applied parallel (0${}^{\circ }$) and perpendicular (90${}^{\circ }$) to the surface of the graphene/Co sample.

The $<L_{z}>$ and $<S_{z}>$ of the Co layer, calculated using the formulae provided in Ref. [22], are 0.057 μ_B_ and 0.21 μ_B_, respectively. Therefore, the total magnetic moment amounts to $m_{total}$ = 0.48 $\mu_{B}$/Co atom, which is lower than previously reported values of ∼1.71 $\mu_{B}$ for bulk Co [36,37,38]. The reduced value is attributed to the fact that the Co magnetisation could not be saturated with an applied field of 0.11 T, as confirmed by the superconducting quantum interference device (SQUID) magnetometry data shown in Figure 5, taken with the magnetic field applied in-plane (0${}^{\circ }$) and out-of-plane (90${}^{\circ }$) to the sample surface. The shape of the hystereses and the size of the coercivities of both measurements show that the magnetic easy axis is in-plane, whereas the reduced sample moments measured by the SQUID are at least in part due to the error arising from measuring the sample’s dimensions accurately.

An upper limit to the orbital momentum of the graphene layer was estimated by the integral of the modulus of the dichroic signal, $|\mu_{XMCD}|$, represented by the red curve in Figure 4c. Despite the reduced dichroic contrast, and thus the magnetic moment, for the Co film the XMCD signal of the C *K*-edge (see Figure 4c) gives a clear hint that a magnetic moment has been induced into the graphene layer.

The upper bound value for the orbital magnetic moment, $m_{o}$, value was calculated to be 0.012 μ_B_/C atom for the C *K*-edge by using the number of holes to be $n_{h}=4$. Due to the spherical symmetry of the initial *s* state, the *s* to *p* transition allows us only to estimate the orbital moment. To estimate the spin moment, we used the relation between the orbital and spin moment in terms of the gyromagnetic factor, *g*, $m_{s}=2m_{o}/\left(g-2\right)$ [39]. The spin magnetic moment, $m_{s}$, is then estimated to be 0.08 $\mu_{B}$/C atom, by using a spin *g*-factor of g=2.3, previously reported for graphene on SiC [40]. Hence, the total magnetic moment, $m_{total}$, of the graphene grown on Co amounts to 0.092 $\mu_{B}$/C atom as estimated by XMCD. If one further accounts for the partial saturation of the Co magnetisation, as compared to the saturated value and assuming a linear increase in the magnetic moment of the graphene, we estimate an upper limit for the graphene magnetic moment of $\mu_{total}=0.092\times \frac{1.71}{0.48}=0.33\;\mu$_B_/C atom. The higher source of error in using this method to estimate the induced magnetic moment is discussed in Ref. [22].

### 3.5. Polarised Neutron Reflectivity

**Figure 6:**
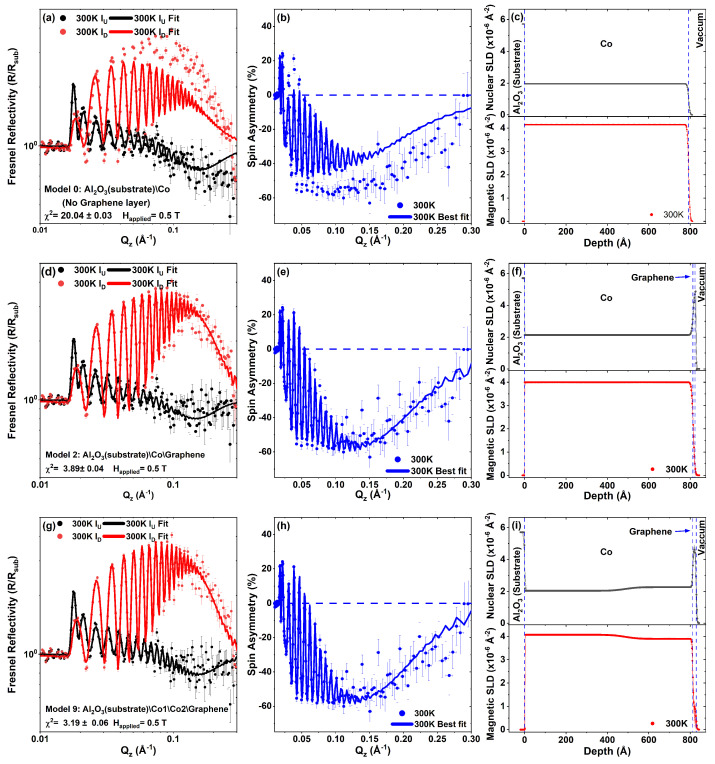
PNR data and results of three fitting models: Here, (**a**,**d**,**g**) show the Fresnel reflectivities at 300 K, (**b**,**e**,**h**) the associated spin asymmetries (SA) and (**c**,**f**,**i**) the nuclear scattering length density (nSLD) profiles (upper panel) and the magnetic scattering length density (mSLD) profiles (lower panel).

The PNR data for the sample collected at 300 K is shown in Figure 6 together with three representative model fits to the data (intermediate 0, 2 and final model 9). The data for the Co/Graphene sample was fitted following the methodology described in Ref. [22]. Panels (a, d and g) of Figure 6 are the Fresnel reflectivities at 300 K, (b, e and h) are the associated spin asymmetries (SA), which scale with the magnetic moment of the sample and (c, f and i) are the nuclear scattering length density (nSLD) profiles (upper panel) and the magnetic scattering length density (mSLD) profiles (lower panel).

Model 0 displayed in panels (a, b and c) is the simplest case of just a single Co layer on an Al${}_{2}$O${}_{3}$ substrate. The magnetism in the Co layer extends over the whole Co layer thickness, with no dead layers or magnetic roughness. It is plain to see that the best fit obtainable does not fit the data. It requires the addition of a non-magnetic-graphene layer on the surface as displayed in panels (d, e and f) for Model 2, confirming the requirement for a graphene layer near the surface.

In order to obtain a good fit to the data, such that it was possible to test whether the graphene layer was magnetic or not, it was necessary to split the Co layer into two regions, as shown by model 9 displayed in panels (g, h and i) of Figure 6, generating a nuclear scattering length density profile (nSLD) profile shown in panel (i) that is less dense near the substrate and denser near the interface with the graphene. The top Co layer approaches the bulk nSLD value for Co (2.265 × 10${}^{-6}$ Å${}^{-2}$). The magnetism across the two Co regions, shown by the magnetic scattering length density (mSLD) in the lower section of panel (i), has the same, but inverted, pattern with a slightly higher magnetic moment near the substrate and lower magnetic moment near the graphene interface, besides a magnetic dead layer at the interface with the sapphire substrate. The graphene layer thickness is approximately four to five mono-layers thick, in agreement with the Raman and SEM results. The value for the induced moment in the graphene layer remains non-zero, since the 95% Bayesian uncertainty range approaches but does not cross the zero. However, to make the roughness of the graphene conformal to the substrate it was required that it is coupled to that of the underlying Co layer, as was the case for the Ni/Graphene in Ref. [22]. Uncoupling these parameters results in non-physical fits.

During the fitting procedure, the value of the magnetic moment in graphene was allowed to go negative, with the upper and lower bounds set to the value for bulk saturated Co (M${}_{Sat}$ = 1.71 $\mu_{b}$/atom ≈ mSLD = 4.113 × 10${}^{-6}$ Å${}^{-2}$).

The PNR fits estimate a total thickness and the magnetic moment of the Co film to be 81.3 nm and 1.75 $\mu_{B}$/Co atom, respectively, and 1.62 nm for the graphene with a magnetic moment of 0.49 $\mu_{B}$/C atom. The thickness of graphene is estimated to range from 1.33 nm to 1.84 nm, which corresponds to 4–6 layers of graphene, based on a graphite lattice constant of 0.335 nm, consistent with our SEM and Raman data (Table 2).

Based on the XMCD and PNR results, we find that although Co has a higher magnetic moment compared to Ni a similar magnetic moment is induced in the graphene whether grown on Co or as rotated and epitaxial graphene on Ni [22]. This can be attributed to the larger lattice mismatch between the graphene and Co (−1.6%) than to the Ni surface (−1.2%) and the slightly longer bond length between the Co-C (2.16 Å) compared to the case in Ni-C (1.9 Å) [15,17,18,23,41] which counterbalance the stronger magnetic moment of the Co and thus induces a similar magnetism in the top graphene layer as for Ni. As our graphene layer is a mixture of single and multi-layer growth, we will average out the magnetic information over the sample surface with PNR which means that we are averaging over regions that have fewer layers. Similarly, due to the beam size in the XMCD set-up we cannot separate effects from single or multi-layer graphene. This also implies that if the induced magnetic moment in the carbon atoms should be concentrated or higher at the interface we will average it out over the whole graphene thickness. We also stress that PNR fitting is highly model dependent (see Aboljadayel et al. [22] for a run through the fitting methodology as to why this model gives a good estimate courtesy of the Bayesian evidence term [42]). Nonetheless, some caution should be taken with the precision of the Bayesian uncertainty ranges, in that they are correct for this fitting model, which gives an excellent fit to the data, but may not capture the data in its entirety.

## 4. Conclusions

To conclude, we have grown multilayer rotated graphene (≈5 layers) by CVD on a highly textured hcp Co(0001) substrate. An induced magnetic moment in the graphene due to the vicinity to the Co film was measured by element-specific XMCD measurements at the C *K*-edge, while the application of the sum rules yielded an estimated magnetic moment of ≈0.33μ_B_/C atom. Additional PNR experiments were performed to confirm the finding and to determine more accurately the magnitude of the magnetic moment detected. The PNR results indicate that the rotated graphene film on Co had a magnetic moment of ≈$0.49\mu_{B}$/C atom, similar in magnitude to that of rotated- domain graphene on Ni(111) although Co has a higher magnetic moment. This is attributed to the slightly longer FM-C bond length and the larger lattice mismatch between the Co and graphene layer.

## Figures and Tables

**Table 1:** Summary of the XRR results for the rotated graphene/Co structure consisting of sapphire substrate/FM layer split into two regions/graphene. The values in parenthesis are the lower and upper 95% Bayesian confidence limits [29].

Layer	Thickness (z)	Roughness (σ)	SLD (ρ)
	[nm]	[nm]	[×**10**${}^{-6}$**Å**${}^{-2}$]
Graphene	1.86 [1.85, 1.88]	0.64 [0.63, 0.65]	10.5 [10.4, 10.8]
Cobalt 2	1.83 [1.78, 1.89]	0.64 [0.63, 0.65]	52.0 [52.0, 52.1]
Cobalt 1	79.4 [79.4, 79.5]	1.55 [1.51, 1.60]	62.0 [61.8, 62.3]
Sapphire	Substrate	0.02 [0.02, 0.023]	30.3 [30.3, 30.32]

**Table 2:** Summary of the PNR results for the rotated graphene/Co, from model 9: sapphire/FM layer split into two regions/graphene. The values in the parenthesis are the lower and upper 95% Bayesian confidence limits [29].

	FM Layer1 + FM Layer2			Graphene	
Sample	Temperature	Total Thickness	Magnetic Moment	Thickness	Magnetic Moment
	[K]	[nm]	[$\mu_{B}$/atom]	[nm]	[$\mu_{B}$/atom]
Gr/Co	300	81.3(81.1,81.4)	1.75(1.73,1.77)	1.62(1.33,1.84)	0.49(0.09,0.62)

## Data Availability

The data is available at the following DOI’s https://doi.org/10.5286/ISIS.E.RB1510330 and https://doi.org/10.5286/ISIS.E.RB1610424.
